# Enhancing the light-driven production of d-lactate by engineering cyanobacterium using a combinational strategy

**DOI:** 10.1038/srep09777

**Published:** 2015-05-05

**Authors:** Chao Li, Fei Tao, Jun Ni, Yu Wang, Feng Yao, Ping Xu

**Affiliations:** 1 State Key Laboratory of Microbial Metabolism, and School of Life Sciences & Biotechnology, Shanghai Jiao Tong University, Shanghai 200240, People’s Republic of China

## Abstract

It is increasingly attractive to engineer cyanobacteria for bulk production of chemicals from CO_2_. However, cofactor bias of cyanobacteria is different from bacteria that prefer NADH, which hampers cyanobacterial strain engineering. In this study, the key enzyme d-lactate dehydrogenase (LdhD) from *Lactobacillus bulgaricus* ATCC11842 was engineered to reverse its favored cofactor from NADH to NADPH. Then, the engineered enzyme was introduced into *Synechococcus elongatus* PCC7942 to construct an efficient light-driven system that produces d-lactic acid from CO_2_. Mutation of LdhD drove a fundamental shift in cofactor preference towards NADPH, and increased d-lactate productivity by over 3.6-fold. We further demonstrated that introduction of a lactic acid transporter and bubbling CO_2_-enriched air also enhanced d-lactate productivity. Using this combinational strategy, increased d-lactate concentration and productivity were achieved. The present strategy may also be used to engineer cyanobacteria for producing other useful chemicals.

Due to heavy use of fossil resources, atmospheric CO_2_ levels have increased approximately by 25% during the past 150 years^1^. The increased CO_2_ level has greenhouse effects and has altered the emissions of methane and nitrous oxide, which have a much higher global warming potential than CO_2_^2^. Because of these concerns, biomass resources such as sugars are considered as the major substitutes for fossil resources^3^. However, using biomass as fossil substitutes leads to direct competition for resources between energy and food supplies. Therefore, it is necessary to develop biosynthetic processes which don’t need to use edible biomass as feedstocks. Direct conversion of CO_2_ to biofuels and carbohydrates using photoautotrophic organisms such as cyanobacteria can resolve the issues regarding both CO_2_ emission and resources shortage simultaneously^1^.

d-Lactate, an isomeric form of lactate, is used as basic feedstock of biodegradable polylactide, a well-known sustainable bioplastic material with lots of commercial applications^4^. d-Lactate is a chiral chemical, which is also used in the pharmaceutical industry and as a precursor for industrial chemicals such as cosmetics^5^. Moreover, its ester derivatives can be used to produce perfumes, coatings, adhesives, and printing ink, and have applications in the electronics industry^5^,^6^,^7^. Generally, d-lactate is produced by lactic acid bacteria from foods containing hexoses and pentoses or from sugar-containing raw materials^7^,^8^,^9^. To reduce utilization of food-related biomass resources, it is necessary to design cell factories that can directly use CO_2_ as the carbon source. More importantly, the cell factories consume CO_2_, thereby relieving material shortage and retarding climate change^1^,^10^. As lactate biosynthesis in cyanobacteria is attractive, several studies have investigated lactate production in cyanobacteria^11^,^12^,^13^,^14^,^15^,^16^,^17^,^18^. However, engineering of cyanobacteria for d-lactate production is limited by relatively low productivity, although efforts have been made to enhance its biosynthesis through both genetic engineering and optimization of culture conditions^14^,^16^,^17^. Low productivity may be attributed to cofactor imbalance (insufficient supply of NADH), because cyanobacteria produce NADPH as the major carrier of reducing equivalents^19^. This hypothesis was confirmed by previous studies where soluble transhydrogenase (*sth*) was introduced into cyanobacteria during d-lactate synthesis^11^,^16^,^17^. Other studies using native NADPH-dependent enzymes were successful with high titers of target products^3^,^20^,^21^,^22^,^23^. However, considering that bacterial NADH-dependent oxidoreductases are more abundant than NADPH-dependent ones, it would be interesting to reverse coenzyme specificity using protein engineering^24^,^25^,^26^,^27^,^28^,^29^. Recently, Angermayr *et al*.^13^ reported that genetically engineered l-lactate dehydrogenase (coenzyme specificity changed from NADH to NADPH) resulted in increased l-lactate production in *Synechocystis* sp. PCC6803. Therefore, rational creation of efficient d-lactate dehydrogenase features a high preference for NADPH, and it is promising to utilize NADPH in cyanobacteria for d-lactate production.

In this study, a combinational strategy was used for construction of a cyanobacterial strain for d-lactate production. Firstly, the comparison of cyanobacterial genomes was performed. Then *Synechococcus elongatus* PCC7942 was selected as the host strain because it lacks l- and d-lactate dehydrogenases, ethanol dehydrogenase, and formate-lyase genes (GenBank ID, CP000100), and could grow to high density within enclosed bioreactors^30^. Secondly, the coenzyme specificity of the key d-lactate producing enzyme, LdhD, was switched from NADH to NADPH by protein engineering. Thirdly, codon usage of LdhD was optimized. Furthermore, as photoautotrophs, cyanobacteria generally lack transporters to move hydrophilic organic molecules across cell membranes^31^. Therefore, a lactate transporter was integrated into *S. elongatus* PCC7942. Finally, CO_2_ bubbling was used to enhance d-lactate production by the constructed *S. elongatus* strain.

## Results

### Switching the coenzyme specificity of LdhD

Bacteria are important gene sources for cyanobacterial engineering. For example, bacteria possess a large number of NADH-dependent oxidoreductases, which are crucial enzymes in metabolic pathways. Unfortunately, the concentration of NADPH in cyanobacteria is much higher than that of NADH^19^; this limits the application of NADH-dependent oxidoreductases in cyanobacteria. A previous study has shown that it is possible to reverse the coenzyme specificity of xylitol dehydrogenase (XDH) from NADH to NADPH using site-directed mutagenesis^25^. To design NADPH-dependent enzymes for cyanobacterial engineering, several key enzymes in bacterial pathways were analyzed. As shown in Table S1, there are sequence gaps around the putative coenzyme binding regions of the first four enzymes, but Asp^176^-Asn^180^ in LdhD and its corresoponding regions in XDH and other enzymes are obviously homologous, indicating that aspartate, asparagine, and the hydrophobic residues are conserved. Therefore, these enzymes may be engineered to utilize NADPH as the preferred cofactor.

D-Lactate is a bio-based chemical that can be produced by fermentation. The key enzyme for d-lactate production in *Lactobacillus bulgaricus* ATCC11842^32^ has the same discriminatory sites between NADH and NADPH (Asp^176^, Ile^177^, Phe^178^, and Asn^180^) as XDH (Table 1). Thus, this enzyme was selected to investigate the applications of LdhD in cyanobacterial engineering. To evaluate the effect of single substitution mutation on cofactor specificity, four single mutants, LdhDn^A^ (D176A), LdhDn^R^ (I177R), LdhDn^S^ (F178S), and LdhDn^R2^ (N180R), were constructed and were expressed in recombinant *Escherichia coli* BL21(DE3) (Table S2). All four single mutations produced positive effects on NADPH kinetics, compared with wild-type LdhD, suggesting that these single substitutions might contribute independently to cofactor reversal in LdhD (Table 2). However, these single mutants still preferred NADH to NADPH.

To further increase the cofactor specificity of LdhD towards NADPH, a quadruple mutant LdhDn^ARSdR^ (D176A/I177R/F178S/N180R) was generated and was expressed in *E. coli* (Table S2). As shown in Table 2, the *k*_*cat*_/*K*_*m*_^NADH^ value of LdhDn^ARSdR^ dropped approximately 28.2-fold compared to LdhD; the *K*_*m*_ of the enzyme for NADH increased while the *k*_*cat*_ decreased. Interestingly, *k*_*cat*_/*K*_*m*_^NADPH^ was approximately 5.2-fold higher than *k*_*cat*_/*K*_*m*_^NADH^, suggesting that there might be a synergistic effect in the quadruple mutant, leading to improved catalytic efficiency for NADPH. Although the *k*_*cat*_/*K*_*m*_^NADPH^ value of LdhDn^ARSdR^ did not reach the *k*_*cat*_/*K*_*m*_^NADH^ value of LdhD, the mutations drove a fundamental shift in cofactor preference toward NADPH. Furthermore, the kinetic constants for pyruvate were measured (Table 2). *K*_*m*_^pyruvate^ with NADH was 1.1 ± 0.1 mM for wild type LdhD and 10.3 ± 0.5 mM for LdhDn^ARSdR^; *K*_*m*_^pyruvate^ with NADPH was 2.25 ± 0.2 mM for LdhDn^ARSdR^. This result suggested that the *K*_*m*_ for pyruvate had not changed significantly. Moreover, the catalytic activity of LdhDn^ARSdR^ did not decrease after incubation at 30 °C for 24 h, suggesting that the enzyme is stable (data not shown).

### Construction of d-lactate-producing *S. elongates* strains

**Figure 1:**
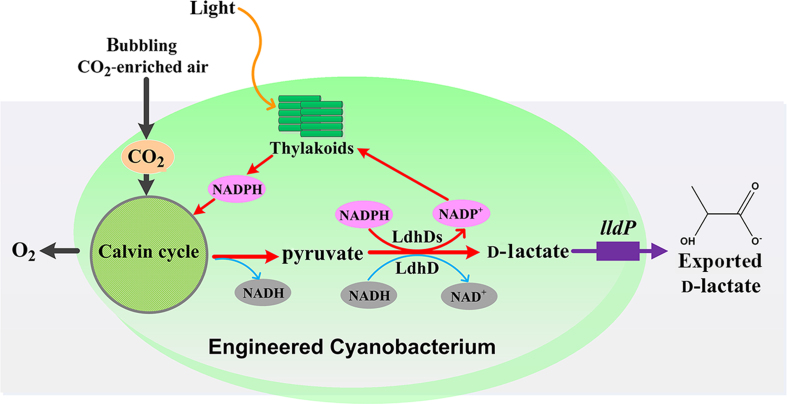
**Engineering strategy for enhancing light-driven production of d-lactate in *S. elongates* PCC7942.** A combinational strategy is taken into account: (i) the engineered *S. elongates* PCC7942 contains *ldhDs* for the conversion of pyruvate to d-lactate (the traditional lactate synthesis pathway is in blue; alternative route with recycling of NADPH is in red); (ii) a lactate transporter encoded *lldP* transports d-lactate extracellularly; and (iii) bubbling CO_2_-enriched air into the culture medium. *ldhDs*, represents *ldhD*, *ldhDc*, *ldhDn*^*ARSdR*^ and *ldhD*^*ARSdR*^.

**Figure 2:**
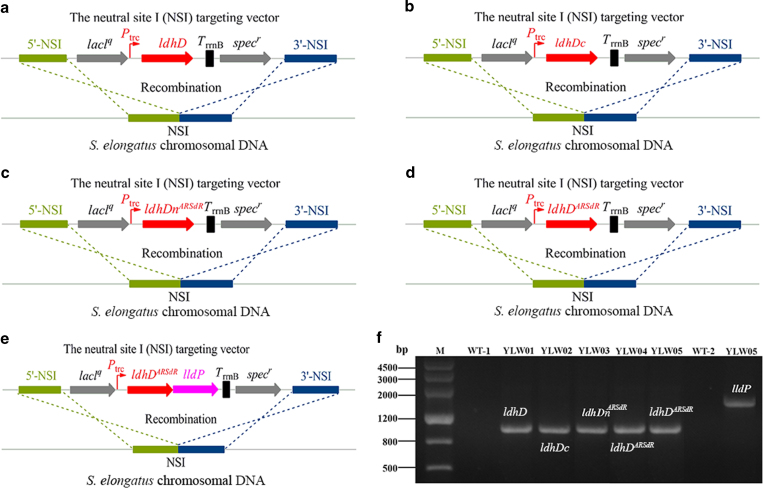
**Construction of** d**-lactate-producing S. elongates strains**. (**a**-**e**) The integration of genes *ldhD, ldhDc, ldhDn^*ARSdR*^, ldhDARSdR and lldP* into the chromosome of *S. elongates* PCC7942. (**f**) Polymerase chain reaction (PCR) confirmed the integration of each gene into the genomic DNA of mutants YLW01, YLW02, YLW03, YLW04 and YLW05. The wild-type strain PCC7942 was used as controls. Cropped gel/blot is used in (**f**).

The biosynthetic pathway of d-lactate uses pyruvate, a central metabolic intermediate that can be reduced to lactate. To engineer *S. elongatus* PCC7942 for d-lactate production, LdhDn^ARSdR^ was then introduced into the strain to facilitate direct utilization of the NADPH pool (Fig. 1). The original enzyme, LdhD, was used to construct a control strain (Fig. 1). To enhance expression in *Synechococcus*, codon-optimized versions of the above two genes, termed as *ldhDc* and *ldhD*^*ARSdR*^, were also synthesized (Table S2). These four genes were all expressed under the control of the IPTG-inducible promoter, *P*_*trc*_ (Fig. 2a–d; Table S3). To further enhance d-lactate production, LldP, a lactate transporter, was expressed under the same promoter (Fig. 2e; Table S3). Neutral site I of *S. elongates* PCC7942 chromosome was used to integrate the cassettes that contained in the constructed plasmids pYLW11, pYLW12, pYLW13, pYLW14, and pYLW24. The resulting strains were named as YLW01, YLW02, YLW03, YLW04, and YLW05, respectively (Table S3). Integration of the inserted genes into the chromosome was verified with PCR and DNA sequencing (Fig. 2f).

### d-Lactate production from CO_2_ by *S. elongates*

To determine the optimal IPTG concentration required for d-lactate production, all the engineered *Synechococcus* strains were grown in the presence of 0.1, 0.5, 1, and 2 mM of IPTG. d-Lactate yields were highest at 1 mM IPTG for strains YLW01, YLW02, YLW03, YLW04, and YLW05 (titers of d-lactate were 101 ± 5.3, 104 ± 5.7, 362 ± 17.1, 452 ± 18.7, and 798 ± 30.3 mg/L, respectively; Figure S1a). d-Lactate synthesis was reduced when the concentration of IPTG was above 1 mM. Therefore, 1 mM IPTG was chosen as the optimal concentration for all subsequent experiments. In addition, reverse transcription (RT)-PCR was performed to investigate the expression of *ldhD*, *ldhDc*, *ldhDn*^*ARSdR*^, *ldhD*^*ARSdR*^, and *lldP* (Figure S1b). The result revealed that the transcription of all genes was detectable at this IPTG concentration. As controls, the five mutant strains were cultured in the BG-11 medium^20^ in the absence of IPTG. A small amount of d-lactate was detected in all the strains, indicating slight leaky expression of the LdhDs (data not shown). It is notable that the difficulty in obtaining the transformants (YLW03, YLW04, and YLW05) harboring *ldhDn*^*ARSdR*^ and *ldhD*^*ARSdR*^proved to be quite difficult, suggesting that leaky expression of LdhDn^ARSdR^ might have resulted in low growth rate and transformation efficiency.

**Figure 3:**
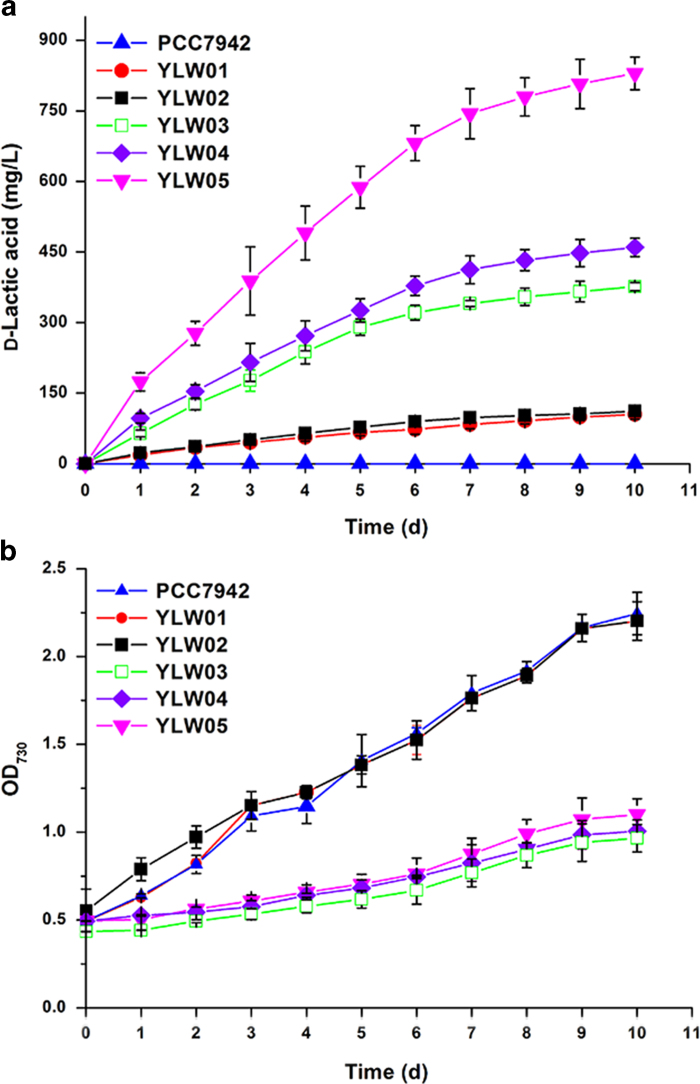
**d-Lactate production by engineered cyanobacteria.** (**a**) Cumulative production of d-lactate by the wild-type 7942 and mutants YLW01, YLW02, YLW03, YLW04, and YLW05 under constant light exposure. (**b**) Time courses for the growth of wild-type PCC7942 and mutants YLW01, YLW02, YLW03, YLW04, and YLW05. Values are the averages of biological replicates; error bars indicate the standard deviations (n = 3); if errors bar are not visible, they are smaller than the respective data point symbol. OD_730_, optical density at 730 nm.

YLW01, YLW02, YLW03, YLW04, and YLW05 were cultured for d-lactate production under constant light exposure (100 μE·s^−1^·m^−2^); the wild type strain *S. elongates* 7942 was used as the control. After induction for 10 days, d-lactate was not detected in the wild type strain (Fig. 3a). Upon introduction of NADPH-utilizing LdhDn^ARSdR^, d-lactate production increased by 3.6-fold and 4.2-fold in YLW03 (37.9 mg/L per day) and YLW04 (46.1 mg/L per day), compared with YLW01 and YLW02 (which harbor native LdhD), respectively (Fig. 3a). The enzymatic activities of the LdhDs in crude *S. elongatus* cell lysate were estimated to confirm the expression of the introduced lactate dehydrogenase genes. As shown in Table 3, although high LdhD activity for NADH was detected in both YLW01 and YLW02, d-lactate production remained low (Fig. 3). This may be attributed to the insufficient concentration of intracellular NADH in cyanobacteria. Conversely, although the activity of LdhDn^ARSdR^ in YLW03 and YLW04 was significantly low, d-lactate production was enhanced in these strains, attributable to the abundant NADPH pool for LdhDn^ARSdR^ in these strains. These results are consistent with a recent report that l-lactate productivity was enhanced by introducing a mutated l-lactate dehydrogenase that could co-utilize NADPH^13^. In addition, to determine the effect of codon optimization on d-lactate production, the relative protein expression profiles of LdhDs were measured. Higher protein levels were observed in YLW02 and YLW04 (~0.80 and ~0.38 × 10^−1^ mg/mg in YLW02 and YLW04, respectively; compared with ~0.56 and ~0.13 × 10^−1^ mg/mg in YLW01 and YLW03, respectively). However, d-lactate production increased only by 1.04- and 1.21-fold in YLW02 and YLW04, compared with YLW01 and YLW03, respectively (Table 2; Table 3). This indicated that codon optimization of *ldhD* and *ldhDn*^*ARSdR*^ increased d-lactate production only marginally. Overall, our results reinforce the importance of using cofactor-altered LdhDn^ARSdR^ for d-lactate production in *S. elongatus* PCC7942.

It is notable that the growth rate of strains containing LdhDn^ARSdR^ was different from that of other strains. There was no significant difference in cell growth among the wild type, YLW01, and YLW02 strains, which had not reached stationary phase at the tenth day and seemed to be able to continue (Fig. 3b). On the other hand, the cell growth rate of strains YLW03, YLW04, and YLW05 was impaired (maximum OD_730_ values of 0.95, 0.99, and 1.07, respectively). A similar phenomenon was also observed in previous reports in which a soluble transhydrogenase or NADPH co-utilizing l-lactate dehydrogenase was introduced^11^,^13^ (Fig. 3b).

The hydrophobic cell membrane is the main barrier for the production and secretion of hydrophilic products such as lactate by genetically engineered cyanobacteria^33^. An l-lactate transporter, LldP, has been described as a nonspecific d-lactate transporter that efficiently transports d-lactate by using proton motive force in *E. coli* and cyanobacteria^16^,^34^. With the expression of the additional *lldP* gene, YLW05 secreted 829 mg/L of d-lactate in 10 days with an average productivity of 82.9 mg/L per day (Fig. 3a). Although the activity of LdhDn^ARSdR^ was lower in YLW05 (compared with that in YLW04), probably because of downregulation of the two genes upon co-expression (Table 3), d-lactate titer in YLW05 was approximately 1.8-fold higher than that in YLW04. This indicates that the transporter efficiently translocated lactate in YLW05, thereby improving d-lactate productivity.

### Effect of aerating CO_2_ on d-lactate production

**Figure 4:**
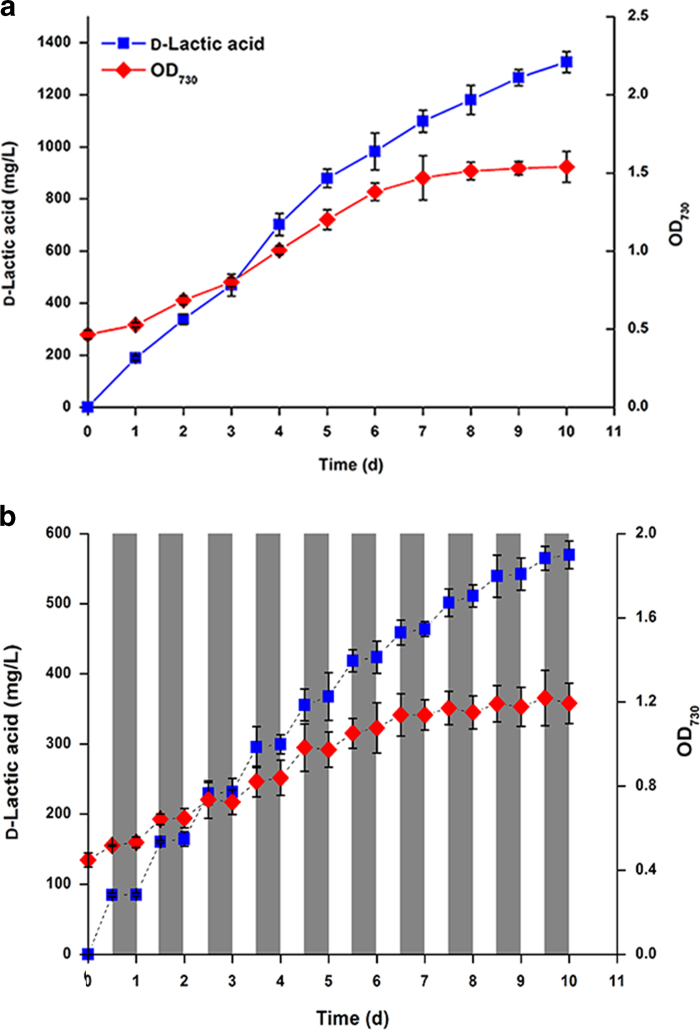
**Effect of aerating CO_2_ on d-lactate productivity of *S. elongates* YLW05.** (**a**) Concentration of secreted d-lactate and growth curve of *S. elongates* YLW05 under constant light exposure. (**b**) Concentration of accumulated d-lactate and cell density of YLW05 under alternating dark and light periods. The gray areas represent the dark periods. Values are the averages of biological replicates; error bars indicate the standard deviations (n = 3); if errors bar are not visible, they are smaller than the respective data point symbol. OD_730_, optical density at 730 nm.

In order to test if lactate production by the *Synechococcus* mutant strain could be enhanced, strain YLW05 was grown in a bubble column photobioreactor by continuously aerating CO_2_-enriched air (5%, v/v; Fig. 4). As expected, aeration with CO_2_ increased d-lactate production in YLW05 (~1.6-fold), reaching a titer of 1.31 g/L in 10 days with a maximum productivity of 221 mg/L per day. Moreover, d-lactate production did not cease after the ninth day, although productivity decreased slightly. As for cell growth, the YLW05 culture with CO_2_-enriched air exhibited slight increase in cell density (Fig. 3; Fig. 4). To further simulate natural production conditions, *S. elongatus* mutant YLW05 was maintained at alternating dark and light periods (at an interval of 12 h) instead of constant light exposure. Under the conditions employed, cell growth was limited to the light period, and cell density decreased slightly in the dark period (Fig. 4b). This result suggests that d-lactate production in *Synechococcus* strains might be promoted by light and inhibited in the dark. This hypothesis is consistent with a previous report stating that cyanobacterial cultures accumulate polysaccharides when they are exposed to light, and they mobilize these intracellular reserve materials in the dark^35^. Finally, strain YLW05 produced a mere 563 mg/L of d-lactate (maximum production rate is 75.1 mg/L per day) after 10 days (Fig. 4b). Nevertheless, these results (productivity of 56.3 mg/L per day) under the day-night cycle conditions suggest that *Synechococcus* strains may be applied to d-lactate production.

## Discussion

Cofactor preference of enzymes is important for microbial organisms to produce metabolites^36^. In this study, to directly use the abundant NADPH pool in cyanobacteria for d-lactate production, a cyanobacterial cell factory was designed by introducing an NADPH-utilizing enzyme, LdhDn^ARSdR^. Significant changes in the kinetic constants of LdhDn^ARSdR^ suggested that the increased d-lactate productivity in YLW03 and YLW04 might stem from the increased activity with NADPH (Table 2). Multiple strategies were tested to optimize lactate production. Altering the cofactor preference of LdhD resulted in over 3.6-fold increase while introducing the transporter, LldP, resulted in approximately 1.8-fold increase, and bubbling CO_2_ resulted in approximately 1.6-fold increase, in d-lactate production. These results show that altering cofactor specificity contributes mostly in enhancing the d-lactate production, which indicates the feasibility of altering the cofactor specificity. In addition, altering the cofactor preference of an existing enzyme has the following possible advantages. First, the cofactor-altered enzyme could directly utilize NADPH, which might be more efficient for product synthesis. Second, compared to the use of a transhydrogenase, it simplifies the metabolic pathways using just one enzyme. Third, altering cofactor specificity might be faster than the process of identifying NADPH-dependent enzymes.

Use of the cofactor-altered LdhDn^ARSdR^ resulted in impaired cell growth. This might be attributed to the high rate of d-lactate production, resulting in decreased NADPH level and activation of the oxidative pentose phosphate cycle. This cycle is the major route of carbon metabolism in cyanobacteria^37^. To confirm this hypothesis, the intracellular levels of NADPH/NADH in both wild type *S. elongatus* PCC7942 and mutant strains were determined during the cultivation process. The ratio of NADPH/NADH slightly decreased in strain YLW04, compared with that in *S. elongatus* PCC7942 (Figure S2). This suggested that the NADPH/NADH ratio altered in the mutant strains, which might have affected the cell growth of strains YLW03, YLW04, and YLW05 (Fig. 3b), respectively. Another possible reason is that redirection of carbon flux from cellular biomass toward synthesis of d-lactate disrupts cell growth. Therefore, the intracellular pyruvate concentration in the wild type and mutant strains was measured. Pyruvate concentration was slightly higher in the wild type strain than that in YLW04 (Figure S3). To overcome this problem of attenuated cell growth, it is necessary to maintain the balance between growth and lactate production by precisely controlling the expression of mutated *ldhD*. This result is also consistent with the above IPTG concentration optimization.

As photoautotrophs, cyanobacteria lack many of the transporters found in *E. coli* or yeast^16^. In two previous studies in which a transporter was introduced into a cyanobacterium for the secretion of lactate, significant improvement in lactate production was observed^15^,^16^. Here, strain YLW05 expressing the *ldhD*^*ARSdR*^ and *lldP* genes secreted relatively high levels of d-lactate into the medium, suggesting that integration of the lactate transporter aids lactate secretion. Moreover, the substrate transport process was mediated by proton translocation, resulting in the accumulation of H^ + ^—a necessary material for the synthesis of NADPH^34^. Therefore, introduction of LldP might contribute to the high yield of d-lactate in strain YLW05 by promoting NADPH production, which can then be used by LdhDn^ARSdR^.

Biosynthesis of d-lactate from CO_2_ has been achieved and is characterized in cyanobacteria, such as *Synechocystis* sp. PCC6803, through genetic engineering^11^,^12^,^13^,^14^,^15^,^16^,^17^,^18^,^32^. As shown in Table S4, both Hollinshead *et al*.^14^ and Varman *et al*.^17^ have reported the enhanced d-lactate production using *Synechocystis* sp. PCC6803 by adding acetate as an organic carbon source. Herein, the concentration and average productivity of d-lactate increased by approximately 90% using *S. elongatus* strain YLW05, compared with strain AV10^14^,^17^, within 10 days. It should be noted that the production in this case was purely photosynthetic. Apparently, the titer values and average productivity of YLW05 were considerably higher than those of other reported strains (10 days), without the addition of an additional carbon source. Based on the above results, it is reasonable to conclude that our combinational strategy for the production of d-lactate might be more effective. Furthermore, to examine whether *S. elongates* PCC7942 was superior, the actual partitioning of carbon between cellular biomass and d-lactate production was evaluated at the late log phase of growth (6 to 8 days for YLW05; 18 to 21 days for AV10). The results revealed that the values for strains YLW05 and AV10 were approximately 80.7 mg/L/OD_730_ and 5.9 mg/L/OD_730_ per day, respectively. This result suggests that strain YLW05 might be more efficient than strain AV10^17^.

In summary, LdhD, a key enzyme in the d-lactate production pathway, was successfully engineered for cofactor reversal, and was used in engineered cyanobacteria for efficient production of d-lactate. Other methods, including introducing a lactate transporter and optimizing codon usage were also adopted in the construction. Under conditions of constant light exposure and bubbling CO_2_-enriched air, the resulting strain (YLW05) achieved the highest lactate concentration and productivity reported for engineered cyanobacteria within 10 days (Table S4). This indicates that the systematic combination of different methods is promising in cyanobacteria engineering. This method of cyanobacterial engineering might have applications in the efficient biosynthesis of other chemicals as well.

## Methods

### Chemicals and reagents

The d-lactate standard, NADH, NADPH, and isopropyl-*β*-d-thiogalactoside (IPTG) were obtained from Sigma-Aldrich (St. Louis, MO). Oligonucleotides and gene synthesis were carried out by Sangon Biotech Co., Ltd. (Shanghai, China). All other chemicals and reagents were of at least analytical grade and were available commercially.

### Strains and growth conditions

*Lactobacillus bulgaricus* ATCC11842 and *Escherichia coli* K-12 strain MG1655 were used as the sources of *ldhD* (GenBank no. 103422405) and the l-lactate transporter gene (*lldP*) (GenBank no. 1790031), respectively. *E. coli* strain DH5α was used as the host for vector construction. *S. elongates* PCC7942 (ATCC33912) was from ATCC (American Type Culture Collection). The *S. elongates* strain was cultured in the BG-11 medium^20^ unless otherwise stated, and cells were incubated statically, at 30 °C and at an illumination intensity of 100 μE·s^−1^·m^−2^, as described elsewhere^14^. Cell growth was monitored by measuring the optical density at 730 nm (OD_730_).

For d-lactate production, *S. elongatus* cells in the exponential phase were diluted to 0.05 (OD_730_) in 100 mL BG-11 medium containing 20 mg/L spectinomycin in 300 mL flasks. Cultures were induced with a suitable concentration of IPTG after growing to an OD_730_ of 0.4–0.6. Daily, 1 mL of each sample was collected for analysis, and equivalent BG11 was supplemented.

### Site-directed mutagenesis and plasmid construction

All primers used for plasmid construction are listed in Table S5. The constructed plasmids are listed in Table S3. A neutral site I (NSI) in *S. elongates* PCC7942 chromosome was used for inserting an expression cassette.

To construct the overexpression vector for the single mutant LdhD^A^ (D176A), the primer pairs ARSR-F/A-R and A-F/ARSR-R (Table S5) were used to amplify the LdhD coding gene from the genomic DNA of *L. bulgaricus* ATCC11842. The PCR products were ligated by splicing with overlapping extension polymerase chain reaction (SOE-PCR)^38^ using primers ARSR-F/A-R and A-F/ARSR-R, and then cloned into the *Bam*HI/*Sac*I site of pETDuet-1, creating pETDuet-*ldhD*^*A*^ (Table S3). Similarly, other single mutants LdhD^R^ (I177R), LdhD^S^ (F178S), and LdhD^R2^ (N180R) were acquired, and cloned into the *Bam*HI/*Sac*I site of pETDuet-1 (Table S5). The resulting plasmids were named as pETDuet-*ldhD*^*R*^, pETDuet-*ldhD*^*S*^, and pETDuet-*ldhD*^*R2*^, respectively (Table S3). To obtain the quadruple mutant (D176A/I177R/F178S/N180R), two pairs of primers (O4_F and C_R, C_F and O4_R) were used to amplify the fragments of *ldhD* from the genomic DNA of *L. bulgaricus* ATCC11842. These two DNA fragments were then ligated by SOE-PCR using primers O4_F and O4_R (Table S5). The resulting gene *ldhDn*^*ARSdR*^ was cloned into pMD18-T for verification by DNA sequencing. Then, *ldhD* and *ldhDn*^*ARSdR*^ were optimized with codon usage (termed as *ldhDc*and *ldhD*^*ARSdR*^, respectively), synthesized with PCR^39^, and also cloned into pMD18-T for sequencing. The primers, O4_F and O4_R, AB_F and AB_R, ARSdR_F and ARSdR_R, were then designed to clone *ldhDn*^*ARSdR*^, *ldhDc,* and *ldhD*^*ARSdR*^, respectively (Table S5).

Two primers, mcs12_F and mcs12_R, were designed for cloning MCS1 and MCS2 fragments (MCS12) from pETDuet-1. For plasmid construction, the PCR product of MCS12 was firstly cloned into the *Eco*RI/*Bam*HI site of plasmid pAM2991^1^ to introduce cloning sites *Afl*II, *Bgl*II, and *Xho*I, creating plasmid pAM-MCS12. The PCR product of *ldhD* was then cloned into the *Eco*RI/*Xho*I site of plasmid pAM-MCS12, creating plasmid pYLW11. Similarly, *ldhDc* was cloned into the *Bam*HI*/Afl*II site of plasmid pAM-MCS12, resulting in plasmid pYLW12. Then, *ldhDn*^*ARSdR*^ and *ldhD*^*ARSdR*^ were cloned into the *Afl*II/*Xho*I site of plasmid pAM-MCS12, resulting in plasmid pYLW13 and pYLW14, respectively. The Shine-Dalgarno (SD) sequence of *ldhD*, *ldhDc*, *ldhDn*^*ARSdR*^ and *ldhD*^*ARSdR*^ was obtained from pET28a( + ).

*lldP* was obtained via PCR amplification from *E. coli* MG1655 using two primers, lldP_3 F and lldP_2 R, it was cloned into the *Xho*I/*Bam*HI site of pYLW14, resulting in plasmid pYLW24. The Shine-Dalgarno (SD) sequence of *lldP* was obtained from *E. coli* MG1655.

### Transformation of *Synechococcus*

Transformation of *Synechococcus* host cells was carried out by using a double homologous-recombination procedure as previously described^16^. Integration of vectors into neutral site I was verified by PCR using gene-specific primers (Table S5) to demonstrate the presence of appropriate novel chromosome-transgene junctions and the absence of uninserted sites. The genetic stability of the mutant strains was evaluated by the serial subcultivation. Table S3 lists the strains that were constructed and used in this study. Briefly, mutant strains were obtained via integrating the aimed DNA fragments harbored by plasmids pYLW11, pYLW12, pYLW13, pYLW14, and pYLW24 to *Synechococcus* chromosome, respectively.

### Culture conditions of *Synechococcus* cells

To investigate the effect of the initial concentration of IPTG on d-lactate production, IPTG concentration was adjusted in the culture media to 0.1, 0.5, 1, and 2 mM. To determine the effect of aeration on d-lactate production, a bubble column photobioreactor equipped with a glass column was used. *S. elongatus* strains were separately suspended in BG11 medium by aerating CO_2_-enriched air under constant light exposure as described in a previous study^10^. To study the effect of the day-night cycle on d-lactate production, *S. elongatus* strain was grown under the aerating condition with day and night periods that alternated every 12 h.

### Enzyme assays

*S. elongatus* cells were harvested via centrifugation (6,000 × *g*, 5 min) 72 h after induction, washed twice with 50 mM Tris-HCl buffer (pH 7.0), and resuspended in the same buffer containing 2 mM dithiothreitol. Crude extracts were prepared via bead beating^22^. Total protein concentration was determined according to the method of Bradford^10^ using bovine serum albumin as the standard. The standard reaction mixture (1 mL) contained 50 mM Tris-HCl buffer (pH 7.0), 0.2 mM NAD(P)H, and 0.05 mM pyruvate. One unit of protein activity was calculated as micromoles of pyruvate consumed per minute per milligram of the total protein at 30 °C.

To characterize the kinetic constants of LdhDn^ARSdR^ after the reversal of coenzyme specificity, the enzyme was expressed in *E. coli* BL21(DE3), with the wild-type LdhD as a control. The purification of the two enzymes were performed using the method of Wang *et al*.^40^ The reduction activity of purified LdhD and LdhDn^ARSdR^ on pyruvate were assayed at 30 °C. Oxidation of NADPH/NADH (ε;340 = 6220 m^−1^ cm^−1^) was monitored by the decrease in absorbance at 340 nm^41^. One unit of protein activity was defined as the amount enzyme that catalyzed the consumption of 1 μmol pyruvate per minute. The reaction mixture (1 mL) contained 50 mM Tris-HCl buffer (pH 7.0), 0.2 mM NAD(P)H, and different concentrations of substrate. The Michaelis-Menten equation was used for determination of the kinetic parameters. To determine the stability of LdhDn^ARSdR^, the purified LdhDn^ARSdR^ were incubated at 30 °C for 24 h.

### Reverse transcription PCR (RT-PCR)

RT-PCR was performed as previously described^21^. Total RNA of the various cyanobacteria strains was extracted using an RNAprep Pure Cell/Bacteria Kit (TIANGEN Biotech Co., Ltd, Beijing, China). RNA was quantified using a NanoVue (GE Healthcare Bio-Sciences AB, Sweden). Residual DNA in RNA preparations was treated with RNase-free DNase I (Thermo Scientific, Shanghai, China). Reverse transcription using random primers was performed with SuperScript^TM^ Ш Reverse Transcriptase (Invitrogen, Shanghai, China). Reverse transcription products were amplified using the specific primers listed in Table S5. The expression of *rnpB* was used as a positive control, and the wild-type *S. elongates* PCC7942 was used as the negative control. PCR products were analyzed with electrophoresis on 2% (w/v) agarose gels.

### Quantification of d-lactate

For d-lactate measurement, 1 mL of the sample was centrifuged (13,000 × *g*, 2 min), cell debris was removed, and the supernatant was boiled for 10 min and centrifuged at 13,000 × *g* for 5 min. The final supernatant was used to determine d-lactate content. Thereafter, d-lactate assay kit (Megazyme) was used to determine d-lactate concentration according to the manufacturer’s instructions^16^. As a control, d-lactate was also assayed in the cell-free supernatant of the wild type strain. Assays were performed in triplicate, and standard deviations were determined.

## Author Contributions

F. T. and P. X. conceived and designed the project and experiments. C. L., F. T., J. N., Y. W., and F. Y. performed the experiments. F. T., C. L., and P. X. analyzed the data. C. L., F. T., and P. X. wrote the paper.

## Additional Information

**How to cite this article**: Li, C. *et al*. Enhancing the light-driven production of D-lactate by engineering cyanobacterium using a combinational strategy. *Sci. Rep*. **5**, 09777; doi: 10.1038/srep09777 (2015).

## Supplementary Material

Supplementary Information

## Figures and Tables

**Table 1:** Partial amino acid sequences alignment of NAD(P)H-binding regions.

Enzyme	Amino acid sequence	
	Site	Coenzyme
XDH^a^	181	VFGAGPVGLLAAAVAKTFGAKGVIVV**DIF**D**N**KLKMAKDIGAATHTFNSK
XDH^ARSdR^ b	181	VFGAGPVGLLAAAVAKTFGAKGVIVV**ARS**D**R**KLKMAKDIGAATHTFNSK
LdhD^a^	150	GVIGTGHIGQVFMQIMEGFGAKVIAY**DIF**R**N**PELEKKGYYVDSLDDLYK
LdhDn^ARSdR^ b	150	GVIGTGHIGQVFMQIMEGFGAKVIAY**ARS**R**R**PELEKKGYYVDSLDDLYK

^a^XDH, xylitol dehydrogenase; LdhD, d-lactate dehydrogenase.

^b^XDH^ARSdR^, cofactor altered xylitol dehydrogenase; LdhDn^ARSdR^, cofactor altered d-lactate dehydrogenase. The coenzyme binding regions are underlined and in bold.

**Table 2:** Kinetic parameters of purified LdhDs for NADH, NADPH, and pyruvate

	Specific activity^a^		Kinetic parameters					
Enzyme	NADH	NADPH	NADH			NADPH		
			*K*_*m*_	*k*_*cat*_	*k*_*cat*_*/K*_*m*_	*K*_*m*_	*k*_*cat*_	*k*_*cat*_*/K*_*m*_
	*unit mg*^−*1*^		*mM*	*s*^−*1*^	*M*^−*1*^*s*^−*1*^	*mM*	*s*^−*1*^	*M*^−*1*^*s*^−*1*^
LdhD	361 ± 29.3	ND^b^	2.3 ± 0.1^*^	561 ± 41.9^*^	(2.4 ± 0.2) × 105 *	ND^*^	ND^*^	ND^*^
			1.1 ± 0.1^**^	441 ± 31.8^**^	(4.0 ± 0.3) × 10^5 **^	ND^**^	ND^**^	ND^**^
LdhD^A^	156 ± 13.2	52.5 ± 4.8	5.9 ± 0.4^*^	190 ± 15.2^*^	(3.2 ± 0.3) × 10^4 *^	6.4 ± 0.7^*^	33.0 ± 2.2^*^	(5.2 ± 0.6) × 10^3 *^
			6.2 ± 0.5^**^	270 ± 21.1^**^	(4.3 ± 0.3) × 10^4 **^	5.9 ± 0.5^**^	3.1 ± 0.4^**^	(1.9 ± 0.1) × 10^3 **^
LdhD^R^	268 ± 21.3	2.7 ± 0.2	3.0 ± 0.2^*^	439 ± 34.8^*^	(1.5 ± 0.1) × 10^5 *^	20.0 ± 1.5^*^	42.8 ± 4.4^*^	(2.1 ± 0.2) × 10^3 *^
			7.2 ± 0.6^**^	1048 ± 89.9^**^	(1.5 ± 0.2) × 10^5 **^	210 ± 20.7^**^	29.9 ± 3.0^**^	(1.4 ± 0.1) × 10^2 **^
LdhD^S^	564 ± 55.8	0.3 ± 0.04	2.7 ± 0.2^*^	500 ± 43.3^*^	(1.9 ± 0.2) × 10^5 *^	23.3 ± 2.1^*^	8.6 ± 0.8^*^	(3.7 ± 0.3) × 10^2 *^
			2.4 ± 0.2^**^	833 ± 65.6^**^	(3.5 ± 0.2) × 10^5 **^	82.5 ± 7.9^**^	21.7 ± 2.0^**^	(2.6 ± 0.2) × 10^2 **^
LdhD^R2^	318 ± 30.2	3.4 ± 0.4	3.0 ± 0.3^*^	594 ± 50.8^*^	(2.0 ± 0.2) × 10^5 *^	29.0 ± 3.1^*^	3.8 ± 0.3^*^	(1.3 ± 0.1) × 10^2 *^
			2.8 ± 0.3^**^	916 ± 92.4^**^	(3.3 ± 0.3) × 10^5 **^	137 ± 9.3^**^	28.8 ± 2.5^**^	(2.1 ± 0.2) × 10^2 **^
LdhDn^ARSdR^	35.0 ± 1.8	150 ± 10.5	15.1 ± 0.8^*^	129 ± 7.6^*^	(8.5 ± 0.3) × 10^3 *^	3.8 ± 0.2^*^	163 ± 12.1^*^	(4.4 ± 0.5) × 10^4 *^
			10.3 ± 0.5^**^	447 ± 35.6^**^	(4.3 ± 0.2) × 10^4 **^	2.25 ± 0.2^**^	335 ± 27.4^**^	(1.5 ± 0.1) × 10^5 **^

^*^Kinetic parameters for NADH or NADPH; ^**^Kinetic parameters for pyruvate.

Values represent mean ± S.D. (n = 3).

^a^Under standard assay conditions as described under “Methods”.

^b^ND, the kinetic parameters were not determined because the enzymes showed no activity towards the substrate.

**Table 3:** Activities of LdhD and LdhDn^ARSdR^ of crude extracts of *S. elongates*.

**Strain**	**Activity**^a^		**Enzyme**
	**NADH**	**NADPH**	
*unit*			
PCC7942	ND^b^	ND	—c
YLW01	201.1 ± 2.3	ND	LdhD
YLW02	289.4 ± 2.6	ND	LdhD
YLW03	0.21 ± 0.02	1.96 ± 0.08	LdhDn^ARSdR^
YLW04	0.68 ± 0.03	5.76 ± 0.25	LdhDn^ARSdR^
YLW05	0.53 ± 0.03	4.34 ± 0.21	LdhDn^ARSdR^

Values represent mean ± S.D. (n = 3).

^a^Under standard assay conditions as described under “Methods”.

^b^ND, the enzymes showed no activity towards the substrate.

^c^Not exist.
