# Supplementary material for: Enhancing the light-driven production of d-lactate by engineering cyanobacterium using a combinational strategy
Source: Sci Rep. 2015 May 5;5:9777. doi: 10.1038/srep09777 (PMC4419521; doi:10.1038/srep09777)
Supplement: Supplementary Information [file srep09777-s1.doc]

**Supplementary Information**

Biotechnology

**Enhancing the light-driven production of d-lactate by engineering cyanobacterium using a** **combinational strategy**

Chao Li, Fei Tao^§^, Jun Ni, Yu Wang, Feng Yao, and Ping Xu

State Key Laboratory of Microbial Metabolism, and School of Life Sciences & Biotechnology, Shanghai Jiao Tong University, Shanghai 200240, People’s Republic of China.

^§^Correspondence: Fei Tao

Mailing address: School of Life Sciences and Biotechnology, Shanghai Jiao Tong University, Shanghai 200240, People’s Republic of China

E-mail: [taofei@sjtu.edu.cn](mailto:taofei@sjtu.edu.cn)

Tel: +86-021-34206647; Fax: +86-021-34206723

Supplementary Methods

**Quantification of pyruvate.** For pyruvate determination, 50 mL of the sample was centrifuged, washed with PBS buffer (50 mM, pH 7.0), and resuspended in the same buffer (1 mL). The cells were boiled for 10 min and centrifuged at 13,000 × *g* for 5 min. The supernatant was used for product analysis. Sample was measured by high-performance liquid chromatography (HPLC, Agilent 1200 series). The system was equipped with a Bio-Rad Aminex HPX-87H column (300 × 7.8 mm) and a refractive index detector. The analysis was performed at 55°C with a mobile phase of 5 mM H_2_SO_4_ at a flow rate of 0.5 mL min^-1^. Assays were performed in triplicate, and standard deviations were determined.

**Determination of** **NADPH and NADH concentrations.** The intracellular concentrations of NADPH and NADH were determined by using EnzyChrom^TM^ NADP^+^/NADPH Assay Kit and EnzyChrom^TM^ NAD^+^/NADH Assay Kit (BioAssay Systems) according to the manufacturer’s instructions. By comparing the slope of absorption to reaction time with standard curves, NADPH and NADH titers were determined and the ratio of NADPH/NADH was calculated thereof. Assays were performed in triplicate, and standard deviations were determined.

**Supplementary Tables**

**Table S1: Partial sequence alignment of dehydrogenases**

| Enzyme^a^ | Accession no. | Source organism | Coenzyme |  | Rossmann-fold^b^ |
| --- | --- | --- | --- | --- | --- |
|  |  |  |  |  | 1 2 3 456 7 |
| LdhD | [CAI96942](http://www.ncbi.nlm.nih.gov/protein/103422405) | *Lactobacillus delbrueckii* | NADH | 151 | VI**G**T**G**HI**G**QVFMQIMEGFGAK-VIAY**DIF**R**N**PE |
| LdhA | ADG85671 | *Escherichia coli* | NADH | 159 | VI**G**T**G**KI**G**VAMLRILKGFGMR-LLAF**DPY**P**S**AA |
| SerA | NP_417388 | *Escherichia coli* | NADH | 156 | II**G**Y**G**HI**G**TQLGILAESLGMY-VYFY**DIE**N**K**LP |
| PdxB | NP_250066 | *Pseudomonas aeruginosa* | NADH | 121 | VV**G**A**G**QV**G**GRLVEVLRGLGWK-VLVC**DPP**R**Q**AR |
| Fdh | BAB41392 | *Staphylococcus aureus* | NADH | 194 | IF**G**F**G**RI**G**QLVAERLAPFNVT-LQHY**DPI**N**Q**QD |
| SDH | EAZ62978 | *Rhodobacter sphaeroides* | NADH | 175 | VM**G**A**G**PI**G**LVTALSALAGGCARVYVT**DLA**P**K**KL |
| XDH | EAZ62959 | *Scheffersomyces stipitis* | NADH | 181 | VF**G**A**G**PV**G**LLAAAVAKTFGAKGVIVV**DIF**D**N**KL |
| LaD | [AF355628](http://www.ncbi.nlm.nih.gov/nuccore/15811374) | *Trichoderma reesei* | NADH | 198 | VC**G**A**G**PI**G**LVSMLCAAAAGACPLVIT**DIS**E**S**RL |
|  |  |  |  |  | 1 2 3 456 7 |

^a^ Ldh, lactate dehydrogenase; SerA, d-3-phosphoglycerate dehydrogenase; PdxB, erythronate-4-phosphate dehydrogenase; Fdh, formate dehydrogenase; SDH, sorbitol dehydrogenase; XDH, xylitol dehydrogenase; LaD, l-arabinitol 4-dehydrogenase.

^b^ 1–3, Gly-X-Gly-X-X-Gly sequence (where X is any amino acid); 4–7, putative coenzyme-recognizing site.

**Table S2: LdhD and its mutations used in this study**

| Name | Relevant characteristics | Reference |
| --- | --- | --- |
| Enzyme |  |  |
| LdhD | The wild-type enzyme from *Lactobacillus* sp. ATCC11842 | (32) |
| LdhD^A^ | LdhD with D176A | This study |
| LdhD^R^ | LdhD with I177R | This study |
| LdhD^S^ | LdhD with F178S | This study |
| LdhD^R2^ | LdhD with N180R | This study |
| LdhDn^ARSdR^ | LdhD with D176A/I177R/F178S/N180R | This study |
| Gene |  |  |
| *ldhDc* | codon-optimized version of *ldhD* | This study |
| *ldhD^ARSdR^* | codon-optimized version of *ldhDn^ARSdR^* | This study |

**Table S3: Strains and plasmids used and constructed in this study**

| Strain or plasmid | Relevant characteristics | Reference |
| --- | --- | --- |
| Strain |  |  |
| *E. coli* DH5α | Commercial transformation host for cloning | Novagen |
| ATCC11842 | *Lactobacillus* sp. ATCC11842, host for cloning the *ldhD* gene | (38) |
| *E. coli* K-12 strain MG1655 | Host for cloning the *lldP* gene and SD sequence | ATCC700926 |
| PCC7942 | *Synechococcus* sp. PCC7942 wild type | ATCC33912 |
| YLW01 | pYLW11, *ldhD* integrated at NSI | This study |
| YLW02 | pYLW12, *ldhDc* integrated at NSI | This study |
| YLW03 | pYLW13, *ldhDn^ARSdR^* integrated at NSI | This study |
| YLW04 | pYLW14, *ldhD^ARSdR^* integrated at NSI | This study |
| YLW05 | pYLW24, *ldhD^ARSdR^*-*lldP* integrated at NSI | This study |
| Plasmid |  |  |
| pETDuet-1 | Used for cloning MCS12 fragment and SD sequence | Novagen |
| pMD18-T | *E. coli* cloning vector, *amp^r^* | Takara Co., Ltd. |
| pETDuet-*ldhD^A^* | *ldhD^A^* in pETDuet-1 | This study |
| pETDuet-*ldhD^r^* | *ldhD^r^* in pETDuet-1 | This study |
| pETDuet-*ldhD^s^* | *ldhD^s^* in pETDuet-1 | This study |
| pETDuet-*ldhD^r2^* | *ldhD^r2^* in pETDuet-1 | This study |
| pAM2991 | P_trc_, MCS, ColE1, *lacl^q^*, NSI targeting, *spec^r^* | (1) |
| pAM-MCS12 | P_trc_, MCS12, ColE1, *lacl^q^*, NSI targeting, *spec^r^* | This study |
| pYLW11 | P_trc_, *ldhD*, ColE1, *lacl^q^*, NSI targeting, *spec^r^* | This study |
| pYLW12 | P_trc_, *ldhDc*, ColE1, *lacl^q^*, NSI targeting, *spec^r^* | This study |
| pYLW13 | P_trc_, *ldhDn^ARSdR^*, ColE1, *lacl^q^*, NSI targeting, *spec^r^* | This study |
| pYLW14 | P_trc_, *ldhD^ARSdR^*, ColE1, *lacl^q^*, NSI targeting, *spec^r^* | This study |
| pYLW24 | P_trc_, *ldhD^ARSdR^*, *lldP,* ColE1, *lacl^q^*, NSI targeting, *spec^r^* | This study |

**Table S4: Comparison of lactate production by various cyanobacterial strains**

| Host organism | Product | Concentration^a^  (mg/L) | Time  (day) | Productivity^b^  (mg/L per day) | Reference |
| --- | --- | --- | --- | --- | --- |
| *Synechocystis* sp. PCC6803 | l-lactate | 288.0 (162.0) | 14 | 20.6 (16.2) | (11) |
| *Synechocystis* sp. PCC6803 | l-lactate | 1800 (180.0) | 28 | 64.3 (18.0) | (12) |
| *Synechocystis* sp. PCC6803 | l-lactate | 836.1 (520.0) | 14 | 59.7 (52.0) | (13) |
| *Synechocystis* sp. PCC6803 | l-lactate | 15.3 (3.6) | 18 | 0.85 (0.36) | (15) |
| *Synechocystis* sp. PCC6803 | l-lactate | 126.0 (108.0) | 11 | 14.7 (10.8) | (18) |
| *Synechocystis* sp. PCC6803 | d-lactate | 1200 (<600.0) | 20 | 60.0 (<60.0) | (14) |
| *Synechococcus* sp. PCC7942 | d-lactate | 54.0 (—) | 4 | 13.5 (—) | (16) |
| *Synechocystis* sp. PCC6803 | d-lactate | 2170 (700.0) | 24 | 90.4 (70.0) | (17) |
| *Synechocystis* sp. PCC6803 | d-lactate | 1060 (—) | 6 | 176.7 (—) | (32) |
| *Synechococcus* sp. PCC7942 | d-lactate | 1310 (1310) | 10 | 131.0 (131.0) | This study |

^a^ [Bracket](javascript:void(0);) represents the concentration of accumulated lactate within 10 days.

^b^ [Bracket](javascript:void(0);) represents the productivity of lactate within 10 days.

(—), Not determined.

**Table S5: Sequences of primers used in this study**

| Gene | Primer name | Sequence (5' to 3') |
| --- | --- | --- |
| MCS12^a^ | mcs12_F | TCATCACCACAGCCAGGATC |
|  | mcs12_R | CGCGGATCCTTTACCAGACTCGAGGGTAC |
| *ldhD^A^* ^a^ | ARSR-F | CCGCGGATCCGAAGAAGGAGATATACCATGACTAAAATTTTTGCTTACGC |
|  | A-R | CTTTTCCAATTCTGGGTTGCGGAAGATGGCGTAAGCGATAACCTTAGCGC |
|  | A-F | GCCATCTTCCGCAACCCAGAATT |
|  | ARSR-R | TGACGAGCTCTTAGCCAACCTTAACTGGAG |
| *LdhD^R^* ^a^ | R-R | CTTCTTTTCCAATTCTGGGTTGCGGAAGCGGTCGTAAGCGATAACCTTAG |
|  | R-F | CGCTTCCGCAACCCAGAATTGGA |
| *ldhD^S^* ^a^ | S-R | GCCCTTCTTTTCCAATTCTGGGTTGCGGCTGATGTCGTAAGCGATAACCT |
|  | S-F | AGCCGCAACCCAGAATTGGAAAA |
| *LdhD^R2^* ^a^ | R2-R | GTAGTAGCCCTTCTTTTCCAATTCTGGGCGGCGGAAGATGTCGTAAGCGA |
|  | R2-F | CGCCCAGAATTGGAAAAGAAGGG |
| *ldhD*^a^ | ldhD_F | GCGGCTTAAGAAGAAGGAGATATACCATGACTAAAATTTTTGCTTA |
|  | ldhD_R | ACCGCTCGAGTTAGCCAACCTTAACTGGAG |
| *ldhDc*^a^ | O4_F | CGCGGATCCAAGAAGGAGATATACCATGACTAAAATTTTTGCTTA |
|  | O4_R | ACGGCTTAAGTTAGCCAACCTTAACGGGAG |
|  | C_F | CTTACGACATCTTCCGCAACCCCGAATTGGAAAAGAAGGG |
|  | C_R | TTCGGGGTTGCGGAAGATGTCGTAAGCGATAACCTTAGCG |
| *ldhDn^ARSdR^* ^a^ | AB_F | GCGGCTTAAGAAGAAGGAGATATACCATGACTAAAATTTTTGCTTA |
|  | AB_R | ACCGCTCGAGTTAGCCAACCTTAACTGGAG |
| *ldhD^ARSdR^* ^a^ | ARSdR_F | GCGGCTTAAGAAGAAGGAGATATACCATGACTAAAATTTTTGCT |
|  | ARSdR_R | ACCGCTCGAGTTAGCCAACCTTAACGGGAG |
| *lldP*^a^ | lldP_3F | TCAGCTCGAGAGGAGACCTGCAATGAATCTCTGGCAACAA |
|  | lldP_2R | CGACGGATCCTTAAGGAATCATCCACGT |
| *ldhD*^b^ | ld_F | AAGATGAGCCTGCGTAAC |
|  | ld_R | TTCGTCCATAGCCTTGTC |
| *ldhDc*^b^ | lDc_F | AAGATGAGCCTGCGTAAC |
|  | lDc_R | TTCGTCCATAGCCTTGTC |
| *ldhDn^ARSdR^* ^b^ | lDn_F | AAGATGAGCCTGCGTAAC |
|  | lDn_R | TTCGTCCATAGCCTTGTC |
| *ldhD^ARSdR^* ^b^ | x_F | AAGATGAGCCTGCGTAAC |
|  | x_R | TTCGTCCATAGCCTTGTC |
| *lldP*^b^ | lldP_F | GATTGTCTGGCTGAAGATG |
|  | lldP_R | GCGAGAAGAAGGTGAATG |
| *rnpB*^b^ | rnpB_F | AGCAAGGTGGAGGGACAAC |
|  | rnpB_R | CGAAGACAGAGGGCAGTTATC |

^a^ These primers were used for gene cloning. Underlined included the SD sequences added.

^b^ These primers were used for RT-PCR analysis.

**Supplementary Figures**


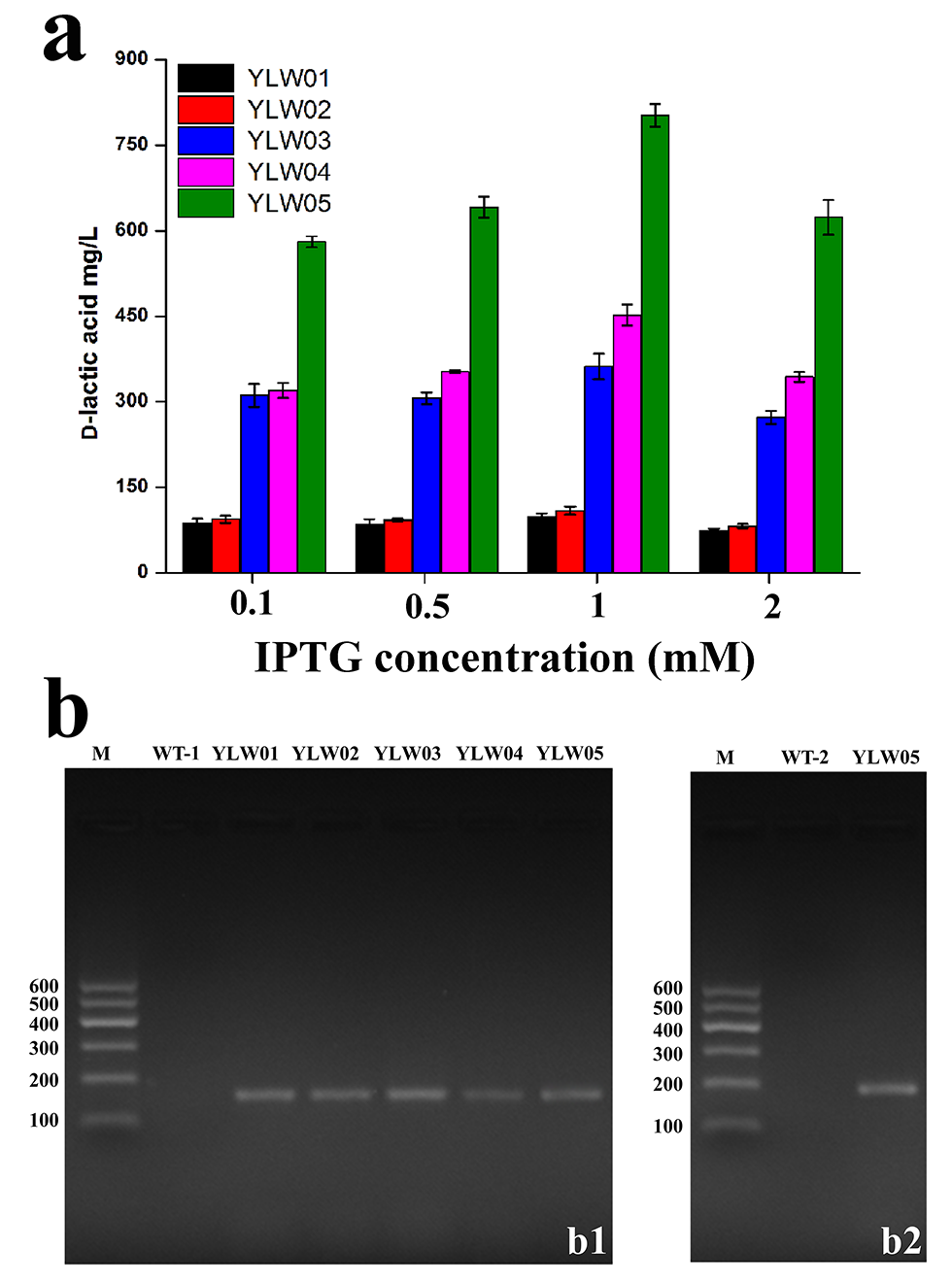


**Figure S1: d-Lactate synthesized by engineered *S.* *elongates* PCC7942 under different isopropyl-*β*-d-thiogalactoside (IPTG) concentrations.** (a) Effect of IPTG concentration on d-lactate production of mutants; (b) Reverse transcription PCR confirmation of expression of heterologous genes *ldhD*, *ldhDc*, *ldhDn^ARSdR^*, *ldhD^ARSdR^* and *lldP* (b1: 165 bp of *ldhDs* from YLW01, YLW02, YLW03, YLW04 and YLW05; b2: 191 bp of *lldP* from YLW05; 1 mM IPTG). M, marker; WT-1 and WT-2, wild-type PCC7942; *ldhDs*, represent *ldhD*, *ldhDc*, *ldhDn^ARSdR^* and *ldhD^ARSdR^*. Values are the averages of biological replicates; error bars indicate the standard deviations (n=3); if errors bar are not visible, they are smaller than the respective data point symbol.


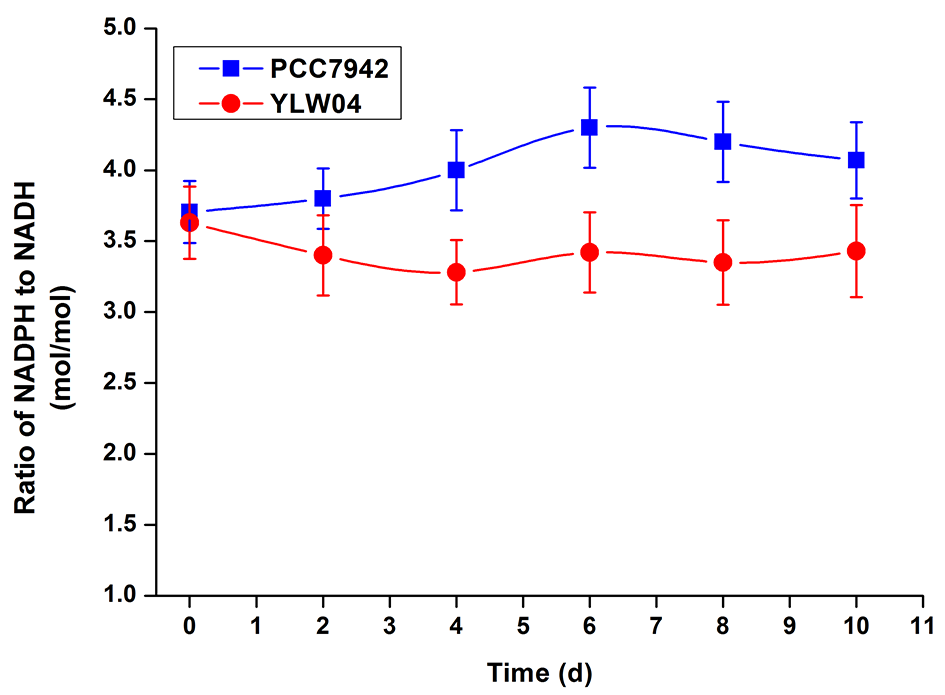


**Figure S2:** **Ratios of intracellular NADPH to NADH in *S. elongatus* PCC7942 and mutant strain YLW04.** Values are the averages of biological replicates; error bars indicate the standard deviations (n = 3); if errors bar are not visible, they are smaller than the respective data point symbol.


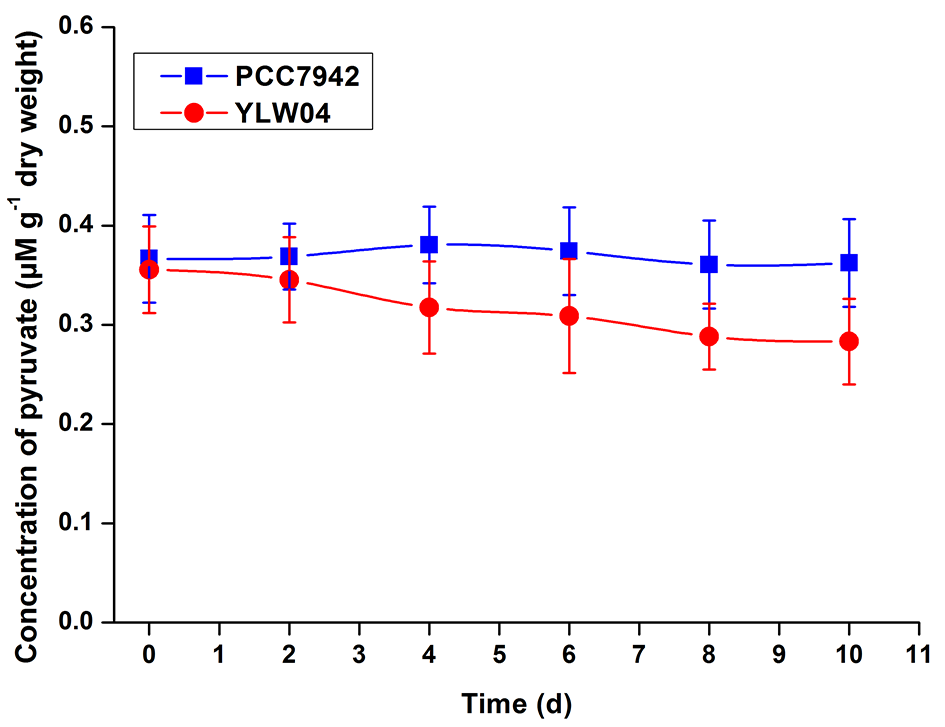


**Figure S3: Concentrations of intracellular pyruvate in *S. elongatus* PCC7942 and mutant strain YLW04.** Values are the averages of biological replicates; error bars indicate the standard deviations (n = 3); if errors bar are not visible, they are smaller than the respective data point symbol.

**Sequence of *ldhDc* (Accession number: KM096560)**

ATGACTAAAATTTTTGCTTACGCAATTCGTGAAGATGAAAAGCCATTCTTGAAGGAATGGGAAGACGCTCACAAGGACGTCGAAGTTGAATACACTGACAAGCTTTTGACCCCAGAAACTGTTGCTTTGGCAAAGGGTGCTGACGGTGTTGTTGTTTACCAACAACTTGACTACACCGCTGAAACTCTGCAAGCTTTGGCAGACAACGGCATCACTAAGATGAGCCTGCGTAACGTTGGTGTTGACAACATCGACATGGCTAAGGCTAAGGAACTTGGCTTCCAAATCACCAACGTTCCAGTTTACTCACCAAACGCCATCGCAGAACACGCTGCTATCCAAGCTGCCCGCATCCTGCGTCAAGACAAGGCTATGGACGAAAAGGTTGCCCGTCACGACTTGCGTTGGGCACCAACTATCGGCCGTGAAGTTCGCGACCAAGTTGTTGGTGTTATAGGTACTGGCCACATCGGTCAAGTCTTCATGCAAATCATGGAAGGCTTCGGCGCTAAGGTTATCGCTTACGCCCGCAGCCGCCGCCCAGAATTGGAAAAGAAGGGCTACTACGTAGACTCACTTGACGACCTGTACAAGCAAGCTGACGTTATTTCCCTGCACGTTCCTGACGTTCCAGCTAACGTTCACATGATCAACGACGAGTCAATCGCTAAAATGAAGCAAGACGTAGTTATCGTTAACGTATCACGTGGTCCATTGGTTGACACTGACGCGGTTATCCGTGGTTTGGACTCAGGCAAGATCTTCGGTTACGCAATGGACGTTTACGAAGGTGAAGTTGGCATCTTCAACGAAGACTGGGAAGGCAAGGAATTCCCAGACGCACGTTTAGCTGACTTAATCGCTCGTCCAAACGTTCTGGTAACTCCACACACTGCTTTCTACACTACTCACGCTGTTCGCAACATGGTAGTTAAGGCCTTCGACAACAACCTTGAATTGGTTGAAGGCAAGGAAGCTGAAACTCCAGTTAAGGTTGGCTAA

**Sequence of *ldhDn^ARSdR^* (Accession number: KM096561)**

ATGACTAAAATTTTTGCTTACGCAATTCGTGAAGATGAAAAGCCCTTCTTGAAGGAATGGGAAGACGCTCACAAGGACGTCGAAGTTGAATACACTGACAAGCTGTTGACCCCCGAAACTGTTGCTTTGGCAAAGGGTGCTGACGGTGTTGTTGTTTACCAACAACTGGACTACACCGCTGAAACTCTCCAAGCTCTCGCAGACAACGGCATCACTAAGATGAGCCTGCGTAACGTTGGTGTTGACAACATCGACATGGCTAAGGCTAAGGAACTGGGCTTCCAAATCACCAACGTTCCCGTTTACAGCCCCAACGCCATCGCAGAACACGCTGCTATCCAAGCTGCCCGCATCCTGCGTCAAGACAAGGCTATGGACGAAAAGGTTGCCCGTCACGACTTGCGTTGGGCACCCACTATCGGCCGTGAAGTTCGCGACCAAGTTGTTGGTGTTATCGGTACTGGCCACATCGGTCAAGTCTTCATGCAAATCATGGAAGGCTTCGGCGCTAAGGTTATCGCTTACGACATCTTCCGCAACCCCGAATTGGAAAAGAAGGGCTACTACGTGGACAGCCTGGACGACCTGTACAAGCAAGCTGACGTTATTTCCCTGCACGTTCCTGACGTTCCCGCTAACGTTCACATGATCAACGACGAGAGCATCGCTAAAATGAAGCAAGACGTGGTTATCGTTAACGTGAGCCGTGGTCCCTTGGTTGACACTGACGCGGTTATACGTGGTCTCGACAGCGGCAAGATCTTCGGTTACGCAATGGACGTTTACGAAGGTGAAGTTGGCATCTTCAACGAAGACTGGGAAGGCAAGGAATTCCCCGACGCACGTCTGGCTGACCTGATCGCTCGTCCCAACGTTCTGGTGACTCCCCACACTGCTTTCTACACTACTCACGCTGTTCGCAACATGGTGGTTAAGGCCTTCGACAACAACCTGGAATTGGTTGAAGGCAAGGAAGCTGAAACTCCCGTTAAGGTTGGCTAA

**Sequence of *ldhD^ARSdR^* (Accession number: KM096562)**

ATGACTAAAATTTTTGCTTACGCAATTCGTGAAGATGAAAAGCCCTTCTTGAAGGAATGGGAAGACGCTCACAAGGACGTCGAAGTTGAATACACTGACAAGCTGTTGACCCCCGAAACTGTTGCTTTGGCAAAGGGTGCTGACGGTGTTGTTGTTTACCAACAACTGGACTACACCGCTGAAACTCTCCAAGCTCTCGCAGACAACGGCATCACTAAGATGAGCCTGCGTAACGTTGGTGTTGACAACATCGACATGGCTAAGGCTAAGGAACTGGGCTTCCAAATCACCAACGTTCCCGTTTACAGCCCCAACGCCATCGCAGAACACGCTGCTATCCAAGCTGCCCGCATCCTGCGTCAAGACAAGGCTATGGACGAAAAGGTTGCCCGTCACGACTTGCGTTGGGCACCCACTATCGGCCGTGAAGTTCGCGACCAAGTTGTTGGTGTTATCGGTACTGGCCACATCGGTCAAGTCTTCATGCAAATCATGGAAGGCTTCGGCGCTAAGGTTATCGCTTACGCCCGCAGCCGCCGCCCCGAATTGGAAAAGAAGGGCTACTACGTGGACAGCCTGGACGACCTGTACAAGCAAGCTGACGTTATTTCCCTGCACGTTCCTGACGTTCCCGCTAACGTTCACATGATCAACGACGAGAGCATCGCTAAAATGAAGCAAGACGTGGTTATCGTTAACGTGAGCCGTGGTCCCTTGGTTGACACTGACGCGGTTATACGTGGTCTCGACAGCGGCAAGATCTTCGGTTACGCAATGGACGTTTACGAAGGTGAAGTTGGCATCTTCAACGAAGACTGGGAAGGCAAGGAATTCCCCGACGCACGTCTGGCTGACCTGATCGCTCGTCCCAACGTTCTGGTGACTCCCCACACTGCTTTCTACACTACTCACGCTGTTCGCAACATGGTGGTTAAGGCCTTCGACAACAACCTGGAATTGGTTGAAGGCAAGGAAGCTGAAACTCCCGTTAAGGTTGGCTAA
